# Molecular characterization of vancomycin-resistant *Enterococcus faecium* isolates from Bermuda

**DOI:** 10.1371/journal.pone.0171317

**Published:** 2017-03-07

**Authors:** Patrick Eberechi Akpaka, Shivnarine Kissoon, Clyde Wilson, Padman Jayaratne, Ashley Smith, George R. Golding

**Affiliations:** 1 Dept. of Paraclinical Sciences, The University of the West Indies, St. Augustine, Trinidad & Tobago; 2 Dept. of Pathology and Microbiology, King Edward VII Memorial Hospital, Hamilton, Bermuda; 3 Dept. of Pathological Sciences, McMaster University, Hamilton, Canada; 4 Dept. of Pharmacy, King Edward VII Memorial Hospital, Hamilton, Bermuda; 5 Public Health Agency of Canada, National Microbiology Laboratory, Winnipeg, Canada; University of Malaya, MALAYSIA

## Abstract

Molecular characteristics of vancomycin resistant enterococci isolates from Bermuda Island is currently unknown. This study was conducted to investigate phenotypic and genotypic characteristics of VRE isolates from Bermuda Island using the chromogenic agar, E-tests, polymerase chain reaction (PCR), pulsed-field gel electrophoresis (PFGE) and multilocus sequence typing (MLST). Eighteen *E*. *faecium* isolates were completely analyzed and were all resistant to vancomycin, susceptible to linezolid and quinupristin/dalfopristin, positive for *van*A and *esp* genes. The MLST analysis confirmed most isolates were of the sequence types linked to clonal complex 17 (CC17) that is widely associated with outbreaks in hospitals. Infection control measures, antibiotic stewardship, and surveillance activities will continue to be a priority in hospital on the Island.

## Introduction

Over the years, vancomycin resistant enterococci (VRE) have become a major threat to public health in many parts of the world. *Enterococcus faecalis* is more common in human infections but vancomycin resistance is more frequently observed in the *E*. *faecium* isolates. The prevalence of VRE in any hospital or health facilities has been associated with higher treatment costs, prolonged morbidity and greater mortality rates [1]. The prevalence of vancomycin resistance amongst enterococci differs widely among different countries including from one hospital to another in the same country [2]. VRE is an important health concern not only because its infections are difficult to treat in healthcare settings but also because the VRE clones can spread within hospitals as well as between regions or countries [3, 2].

The first ever report of vancomycin-resistant *E*. *faecalis* and *E*. *faecium* was in 1988 in the UK [4], and within a decade VRE represented >25% of enterococci associated with bloodstream infections in hospitalized patients in the United States [5]. VRE infection epidemiology as has been reported differs between Europe and the United States and perhaps in many other countries [6]. Nosocomial VRE infection and transmission have occurred much more frequently in the United States and reports have documented, in hospitalized patients, horizontal transfer of the *van*A gene from vancomycin-resistant *E*. *faecalis* to methicillin-resistant *Staphylococcus aureus* (MRSA) [7, 8]

Vancomycin resistant enterococci was first reported in the island of Bermuda in 1995. Subsequently 13 patients were either colonized or infected; and these were all imported cases. No nosocomial transmission and resistance to high level aminoglycosides had then been reported [9]. Since this report was made, there has been no published data of VRE infections, their antimicrobial susceptibility patterns, resistant gene, clones and sequence types in the country, a British island territory in the North Atlantic, off the eastern United States coast with a population of about 65,000. However, there is a policy in place limiting the use of restricted antimicrobials in order to minimize the development of microbial resistance. Vancomycin is among such restricted antimicrobials. The observed hospital associated VRE rate in the country for the period 2007–2015 was less than 0.8 per 1000 patient days.

Molecular epidemiologic studies clarify the genetic relatedness and molecular evolution of VRE clones. In the current study, we investigated the phenotypic and genotypic characteristics of VRE isolates from Bermuda Island by using the chromogenic agar, E-tests, PCR analysis of the *esp* repeat profile, pulsed-field gel electrophoresis (PFGE), multilocus sequence typing (MLST), and PCR to characterize isolates from the country.

## Materials and methods

### VRE isolates

VRE isolates (n = 18) collected between March and December 2013 from patients hospitalized at the King Edward IV Memorial Hospital in Bermuda were analyzed in this study. The source of these isolates were as follows: Urogenital (n = 8 isolates), rectal swab (n = 2 isolates), Blood (n = 2 isolates) and wound swabs (n = 6 isolates). These isolates were from males (n = 4) and females (n = 14), ages between 40 and 79. These VRE isolates were recovered from ICU (n = 5), Medical wards (n = 4) and Surgical ward (n = 11). No duplicate isolates from a single patient were included and there has never been a history of VRE outbreak on the island. These isolates were included in this study if recovered from patients who fulfilled the following criteria: had fever (≥ 38°C), elevated WBC and C-reactive protein. If wound infection is involved, there must be evidence of swelling, redness and tenderness, pus and other systemic symptoms; criteria that has been used in another study elsewhere [10].

#### Patient consent

No consent was obtained from any patients since data were only extracted from patient records and information were anonymized and de-identified prior to analysis.

#### Ethical approval

None was required or obtained from the hospital management. There was no direct contact with any of the patients. Besides information from patients records were anonymized and de-identified and there were no way to trace the bacterial isolates to any of the patients.

### Chromogenic plates detection of vancomycin resistance

The isolates were phenotypically confirmed as VRE by using Chromogenic agar that includes CHROMagar VRE (CHR) medium (CHROMagar, Paris, France) and ChromID VRE (C-ID) medium (bioMerieux, France). All 18 isolates were correctly identified as *E*. *faecium* and these were further subjected to molecular analysis.

### Antimicrobial susceptibility

The susceptibilities for these isolates were determined for vancomycin, ampicillin, levofloxacin, quinupristin/dalfopristin and linezolid by using standard E-test methods. High- level resistance (HLR) to gentamycin and streptomycin was determined using the MicroScan System (Siemesn, USA). The results for all isolates were interpreted according to CLSI guidelines [11]. An MIC ≥16μg/mL for ampicillin and ≥8μg/mL for vancomycin were considered to be resistant.

### Multiplex polymerase chain reaction (PCR)

Determination of glycopeptide resistance genotypes and confirmation of species identification were performed on the isolates by multiplex polymerase chain reaction (PCR), as previously described by Padman et al [12].

### Detection of esp and hyl genes by PCR

The *esp* and *hyl* genes were determined for all isolates by PCR as has been previously described in literature [13, 14]. *E*. *faecium* strain C68 (*hyl*_Efm_ and *esp*_Efm_) was used as the positive control. A 100-bp DNA ladder (Bio-Rad) was used as a molecular size marker

### Repetitive- sequence based PCR (Rep-PCR)

Repetitive-sequence-based PCR (Rep-PCR) methods are rapid typing procedures that amplify the regions between the non-coding repetitive sequences in bacterial genomes [15]. The ERICIR (5’ ATG TAA GCT CCT GGG GAT TCA C-3’) was used for Rep-PCR. The genetic relatedness of VRE isolates was determined by Rep-PCR typing as previously described Healy et al [16]. DNA was extracted using a one μL loop of plated culture or one mL of broth culture and the Ultraclean Microbial DNA isolation kit (Mo Bio Laboratories, Solana Beach, Calif.) following the manufacturer’s instructions.

### Pulsed-field gel electrophoresis (PFGE)

Genomic DNA was prepared in agarose plugs as described by Murray et al and Turabelidze et al with some modifications [17, 18]. After cell lysis by mutanolysin in lysoenzyme and incubation with proteinase, the DNA was digested with *Sma*1. The PFGE was performed using a contour-clamped homogenous electric field apparatus (CHEF DRIII, Bio-Rad Laboratories, Hercules, CA, USA). Gel images were captured on the Gel Doc imaging system using Quality One Software version 4.4.1 (Bio-Rad Laboratories, Hercules, CA, USA). The resulting banding patterns were analyzed by visual inspection according to previously established criteria [19, 20]. Gel analysis was performed using Bionumerics -version 3.5 (Applied Maths, Austin TX, USA) and Cluster analysis was achieved using DICE and UPGMA.

### Multi-locus sequence typing (MLST)

The MLST was carried out with a standard set of primers that amplify the 7 house-keeping genes (*atpA*, *ddl*, *gdh*, *purK*, *gyd*, *pstS*, *adk*) included in the *E*. *faecium* MLST scheme [21, 22]

## Results

The Chromogenic methods were 100% sensitive in detecting all isolates as *E*. *faecium* that were analyzed and used in this study. VRE infections does not conform or adhere to any gender preference or pattern; and is therefore evenly distributed among both sexes. Most of the isolates in our analysis were from women because majority of the patients were women. The antimicrobial susceptibility testing revealed that all isolates were 100% susceptible to Linezolid and quinupristin/dalfopristin. As expected, all isolates were resistant to vancomycin (MIC ≥ 32μg/ml) and ciprofloxacin. But for ampicillin, more than 90% were resistant (MIC ≥ 16μg/ml). As for the high level resistance to the aminoglycosides, 95% were susceptible to gentamycin while only 49% were resistant to streptomycin respectively.

**Fig 1:**
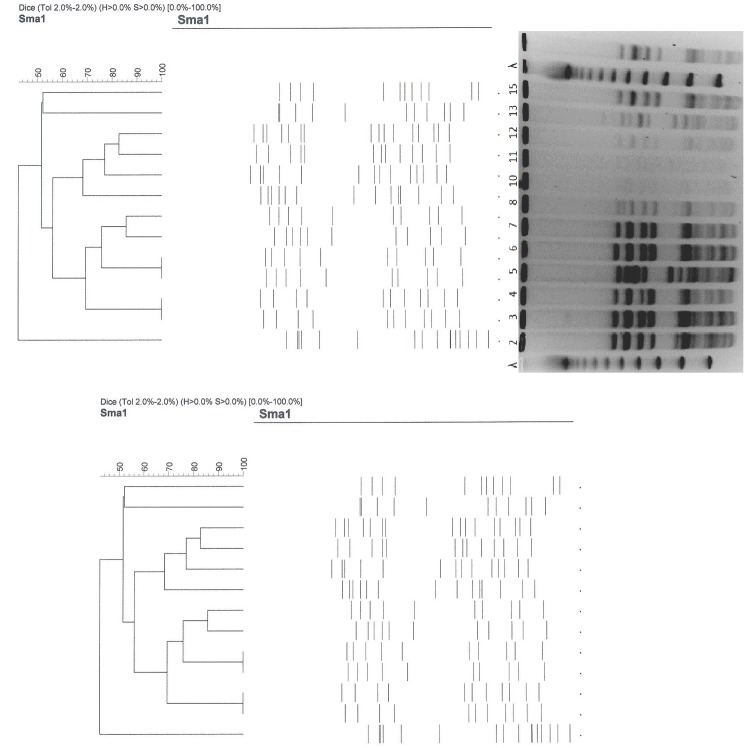
Dendogram of *Sma*1 macro-restriction patterns resolved in PFGE of VREF isolates from Bermuda with the *vanA* genotype.

**Fig 2:**
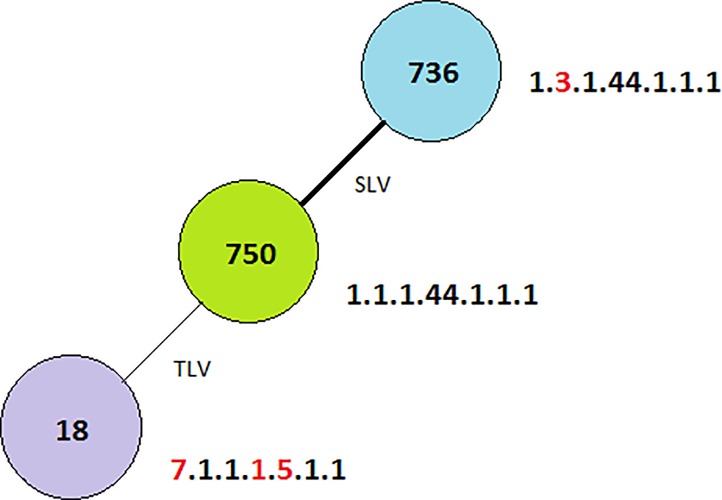
MLST for Bermuda. Darker links represent less allelic differences than lighter links. SLV represents Single Locus Variant while TLV represents Triple Locus Variant.

All analyzed VRE isolates (n = 18) possessed *van*A and *esp* genes but there were no *van*B or *hyl* genes detected. Five different clones were detected by the PFGE method (Fig 1) and these were similar to the *esp* A & C repeats results’ that also varied from three to five pulsotypes. The detection of varied or different clones signifies that the isolates belonged to less than five groups. The MLST analysis revealed three sequence types namely: ST750, ST736 and ST18 as depicted in Fig 2. ST736 is a single locus variant of ST750 (1.3.1.44.1.1.1), while ST18 is a triple locus variant of ST750 (1.1.1.44.1.1.1) → (7.1.1.1.5.1.1) 18. Alterations in the allelic profile compared with ST750 are underlined. Clonal complex 17 was resolved in two subgroups, one of which is ST18.

## Discussion

No study on the molecular characterization of VRE has ever been carried out in Bermuda. And to the best of our knowledge, this is the first report of such activity in the country. In this study, we investigated the genetic characteristics of vancomycin resistant *E*. *faecium* (VREF) isolates from the country by genotypic typing methods including MLST, PFGE, and *esp* repeat profiles. Each method had a different ability to analyze the genotypes of VREF isolates. The MLST identified three different sequence types of VREF isolates in this study, while PFGE revealed five distinguishable fragment patterns. Isolates with different PFGE pattern belonged to the same ST on MLST. This is in agreement with what has already been reported as the usefulness of MLST in determining the long term and evolutionary process of VRE resistant clones, as well as the appropriateness of PFGE in studying the epidemiology of VRE on a short term basis [6, 22]. Both methods proved effective in characterizing the genes and clones prevailing in VRE isolates from Bermuda Island. We also used *esp* repeat profiles to analyze these VREF isolates from Bermuda Island, which showed that isolates from this country belonged to different clones or groups. The diversity of isolates of VRE by the several methods in this study were similar to reports from VRE analysis in Trinidad & Tobago [3]

It is very heartening and encouraging to note that all *E*. *faecium* isolates encountered on the Island are still very susceptible to, linezolid an oxazolidinone antibiotic that is available orally and intravenously; and quinupristine/dalfopristin, a streptogramin combination antibiotic. Linezolid has been specifically approved by the United States Food and Drug Administration for bacteremia cases and it is the most versatile available drugs despite its bacteriostatic activity against VRE and toxicities associated with its prolonged use. Active surveillance on VRE isolates and infections must continue on the Island because although no nosocomial transmission and high level aminoglycosides was reported two decades ago [9], there is evidence of such cases in the current study.

There has never been a report of an outbreak of VRE on the Island despite the observed very low rate of 0.8/1000 patients days over a period of more than 8 years. This is also evident and supported by the paltry number of VRE isolates we were able to isolate from several 900 patients admitted in the hospital during our study analysis (March to December 2013). This number of of isolates may be very insignificant when compared to other hospitals or places that admit several thousands of patients in few months and where the populations is in millions. But for Bermuda that has only one public hospital catering for a population of about 65,000 this number is significant.

In this current study none of the isolates possessed *van*B or *hyl* genes confirming the fact that VRE outbreak is non-existent on the islands as these genes are usually associated with virulence. But the detection of *van*A that is carried on a transposon Tn1546, almost always plasmid-mediated and highly transferable, and *esp* genes among the vancomycin resistant *E*. *faecium* isolates from the country should alert or draw ones attention to report of widespread occurrence and sporadic nosocomial outbreaks caused by such isolates in other places such as USA, Canada, South America and European countries [2, 5, 7, 8, 23–26]. It means that the current high level of infection control measures and antibiotic stewardships practiced in the country should not be relented or compromised.

The DNA profiles of the 18 isolates detected by the PFGE method varied and this finding was similar to the result obtained from the *esp* A & C repeat analysis; and both of these methods depicted a high diversity. This revealed heterogeneous pattern means that several clones of the VRE are circulating on the Island and may not be consistent with multidrug resistance to different antibiotics and the presence of the *van*A gene.

Clustering analysis by the goeBURST algorithm showed that the clonal complexes of the VRE isolates from Bermuda Island were genetically linked to three different sequence types. Majority of these were linked to clonal complex 17. The ST18 was one of the resolved subgroup of clonal complex 17 and it is a major group in the genetic lineage of *E*. *faecium* that is distributed worldwide and has been associated with hospital outbreaks [25]. As previously reported, most linked to this clonal complex are positive for the *van*A and *esp* genes, most are ampicillin and glycopeptides resistance phenotypes, and can thrive and disseminate in the hospital environment [6, 22, 25–27].

## Conclusion

A major limitation in our analysis may be the paltry number of isolates we characterized but despite that, data from this analysis suggests that there was no horizontal spread between strains; and bacterial determinants (*esp*) may not have played a major role in the dissemination of VRE resistance in Bermuda. The genetic lineage of *E*. *faecium* encountered in Bermuda Island belongs to the one distributed worldwide which has been associated with hospital outbreaks. Surveillance, antibiotic stewardship and infection control measures will continue to be a priority on the Island to keep VRE outbreak at bay.
